# Differentially Amplified Repetitive Sequences Among *Aegilops tauschii* Subspecies and Genotypes

**DOI:** 10.3389/fpls.2021.716750

**Published:** 2021-08-19

**Authors:** Rahman Ebrahimzadegan, Fatemeh Orooji, Pengtao Ma, Ghader Mirzaghaderi

**Affiliations:** ^1^Department of Agronomy and Plant Breeding, Faculty of Agriculture, University of Kurdistan, Sanandaj, Iran; ^2^College of Life Sciences, Yantai University, Yantai, China

**Keywords:** satellite repeat, repetitive sequence abundance, tandem repeat, speciation, wheat

## Abstract

Genomic repetitive sequences commonly show species-specific sequence type, abundance, and distribution patterns, however, their intraspecific characteristics have been poorly described. We quantified the genomic repetitive sequences and performed single nucleotide polymorphism (SNP) analysis between 29 *Ae. tauschii* genotypes and subspecies using publicly available raw genomic Illumina sequence reads and used fluorescence *in situ* hybridization (FISH) to experimentally analyze some repeats. The majority of the identified repetitive sequences had similar contents and proportions between *anathera*, *meyeri*, and *strangulata* subspecies. However, two Ty3/gypsy retrotransposons (CL62 and CL87) showed significantly higher abundances, and CL1, CL119, CL213, CL217 tandem repeats, and CL142 retrotransposon (Ty1/copia type) showed significantly lower abundances in subspecies *strangulata* compared with the subspecies *anathera* and *meyeri*. One tandem repeat and 45S ribosomal DNA (45S rDNA) abundances showed a high variation between genotypes but their abundances were not subspecies specific. Phylogenetic analysis using the repeat abundances of the aforementioned clusters placed the *strangulata* subsp. in a distinct clade but could not discriminate *anathera* and *meyeri*. A near complete differentiation of *anathera* and *strangulata* subspecies was observed using SNP analysis; however, *var. meyeri* showed higher genetic diversity. FISH using major tandem repeats couldn’t detect differences between subspecies, although (GAA)_10_ signal patterns generated two different karyotype groups. Taken together, the different classes of repetitive DNA sequences have differentially accumulated between *strangulata* and the other two subspecies of *Ae. tauschii* that is generally in agreement with spike morphology, implying that factors affecting repeatome evolution are variable even among highly closely related lineages.

## Introduction

*Aegilops tauschii* Coss. (2*n* = 2*x* = 14, DD genome) is the D genome progenitor of common wheat (Kihara, 1944; McFadden and Sears, 1946) and the pivotal genome of several polyploid *Aegilops* species (Kimber and Yen, 1988; Mirzaghaderi and Mason, 2017). *Ae. tauschii* harbors a high-genetic variation that can be used in wheat-breeding programs against biotic and abiotic stresses tolerance (Mirzaghaderi and Mason, 2019). Iran is widely referred to as the center of the origin and diversity of *Ae. tauschii* (Dvorak et al., 1998). However, during the long periods of dispersal and adaptation, this species has been distributed through a wide geographical region in the central Eurasia, from the northern Syria and Turkey to the western China (Kihara et al., 1965; Matsuoka et al., 2015).

On the basis of the spike morphology, *Ae. tauschii* has been divided into three varieties, of which two (var. *anathera*, and var. *meyeri*) are grouped into *A. tauschii* subsp. *tauschii*, whereas the third is subsp. *strangulata*. Variety *anathera* is commonly awnless except for the two apical spikelets, while *meyeri* form is very slender and has short spikes with only 4–8 spikelets, awned except the two lowest ones. Subspecies *strangulata* is monophyletic and form a sharply defined moniliform spike. This classification remains controversial because of the presence of intermediate forms so for example var. *typica* has also been mentioned as a distinct variety of subsp. *tauschii* (Eig, 1929; Kihara and Tanaka, 1958; Hammer, 1980; Wang et al., 2013). Variety *meyeri*, which is morphologically an intermediate type between *typica* and *anathera*, is found mainly on the west coast of the Caspian Sea (Kihara et al., 1965).

Interestingly, *Ae. tauschii* botanical classification has a weak agreement with the genetic relationships. Based on the genetic studies, *Ae. tauschii* has been divided into L1 and L2 lineages that are broadly related to *tauschii* and *strangulata* subspecies, respectively (Dvorak et al., 1998; Mizuno et al., 2010). L2 has a limited distribution and is mainly composed of subsp. *strangulata* along with the accessions (mainly var. *meyeri* and a number of accessions from var. *typica*) formerly assigned to subsp. *tauschii* based on spike morphology. These troublesome accessions have likely been originated by the gene migration from subsp. *tauschii* into subsp. *strangulata* (Lubbers et al., 1991; Dvorak et al., 1998). A subpopulation mainly composed of var. *meyeri* and var. *typica* within L2 in the southwestern and southern Caspian appears to be the main source of the wheat D genome (Wang et al., 2013). L1 lineage has been distributed in more diverse environments (Lubbers et al., 1991; Dvorak et al., 1998; Wang et al., 2013).

Understanding the genetic and evolutionary relationships of *Ae. tauschii* accessions might lead to more effective utilization of this species in the wheat breeding (Kilian et al., 2011; Mirzaghaderi et al., 2020). Genomic repetitive sequences commonly show species-specific sequence type, abundance, and distribution patterns, however, there is little information about their intraspecific characteristics. With the increasing genomic data available for the model organisms, it is now possible to investigate repeatome organization among subspecies. Hence, the aim of the present study is to provide an overview of the repetitive sequences in *Ae. tauschii*, and to characterize its dominant lineages related to the botanical classification. Specifically, we analyzed the repetitive sequences of 29 different *Ae. tauschii* genotypes using publicly available low coverage, Illumina-sequencing data, and compared repeat abundance between the different subspecies. The result was further compared to the genome relationships interfered from single nucleotide polymorphism (SNP) analysis and some repeats were localized on the D genome chromosomes using fluorescence *in situ* hybridization (FISH).

## Materials and Methods

### Exploring Repetitive Sequences

Raw Illumina reads (in FASTQ format with 150 bp length) of 29 different *Ae. tauschii* accessions belonging to subspecies *anathera* (10 accessions), *meyeri* (10 accessions), and *strangulata* (9 accessions) (Zhou et al., 2020) (Supplementary Table 1) were downloaded from EBI to RepeatExplorer2 pipeline (Novák et al., 2013, 2020) via Get Data option. Reads were pre-processed using the ‘Preprocessing of FASTQ paired-end reads’ tool using default settings, except that read sampling was set to 500,000 and all the reads were trimmed to 149 nucleotides. Sample codes were added to each sample using “FASTA read name affixer” to specify the corresponding subspecies and accession. All the read samples were merged into a single dataset using “Concatenate datasets tail-to-head” tool. Comparative analysis of repetitive sequences were done by similarity-based clustering of Illumina paired-end reads using the “RepeatExplorer2 clustering” tool (Novák et al., 2020) where 0.01% cluster size threshold (considering only repeats with at least 0.01% of the input reads) and “automatic filtering of abundant satellite repeats” were selected. In the output cluster table, all the clusters were checked manually, and the automated annotation was corrected if needed. The clusters were used to characterize and quantify the most abundant repeats and genomic proportions of the major repeat types were calculated based on the proportion of reads in individual-annotated clusters.

The previously published genome size of 4,968 Mbp per 1C-value (Ozkan et al., 2003) was considered to normalize the sizes of resulting repeat clusters of all the *Ae. tauschii* accessions using optparse package of R version 4.0.2 (The R Project for Statistical Computing, Vienna, Austria) as described in Novák et al. (2020). This generated a plot of rectangles proportional to the amounts of repeats in the genome of the analyzed accessions.

Separate analyses of read samples from each accession were run on RepeatExplorer, using default settings (i.e., similarity threshold of 90 over 55% of the read length) and consensus sequences of the identified repeat monomers were reconstructed by TAREAN (TAndem REpeat ANalyzer) (Novák et al., 2017).

### Phylogenetic Analysis Based on the Identified Repeats

Repeat counts for each genotype were obtained from the output table of the comparative analysis in RepeatExplorer2. Repetitive sequence clusters that differentially amplified between subspecies were identified by the ANOVA. Repeats that showed high-variable abundances between individuals were identified by inspecting the comparative analysis output table manually. A UPGMA (average linkage) tree of 29 *Ae. tauschii* accessions was generated based on the Euclidean distances between the abundances of the repetitive sequence clusters that showed differential amplification between genotypes and subspecies as inferred from RepeatExplorer2. Also, 18S and 26S rDNA genes of the identified 45S rDNA were searched by RNAmmer (Lagesen et al., 2007) and “+” strand of the rDNA sequences of all genotypes were obtained using Range Extractor DNA at https://www.bioinformatics.org/sms2/range_extract_dna.html (Stothard, 2000). Subsequently, the ITS1-5.8S-ITS2 region was extracted for each genotype and used as input for multiple sequence alignment by MUSCLE method using the “msa” package (Bodenhofer et al., 2015). A phylogenetic maximum likelihood tree was obtained using the “phangorn” package (Schliep, 2010) with 100 bootstrapping replications. Box plots were generated based on read abundances in R using ggplot2 package.

### Characterization of Transposable Element

Identification and classification of transposable element protein domain sequences were performed using the DANTE tool at https://repeatexplorer-elixir.cerit-sc.cz/galaxy/(Novak et al., 2019) and the REXdb database (Neumann et al., 2019). The output gff3 files belonging to the different retrotransposons were used to generate corresponding bed and bedgraph files that were subsequently visualized in the Integrative Genomics Viewer (IGV) software (Robinson et al., 2017).

### Variant Calling, Quality Control of SNPs, and Genetic Diversity Analysis

The same sequence reads of *Ae. tauschii* accessions that used for the above mentioned RepeatExplorer analysis, were also downloaded and mapped to their corresponding reference genome (Aegilops_tauschii.Aet_v4.0.dna_rm.toplevel.fa) with Bowtie2 (Langmead and Salzberg, 2012) after sequence trimming with Trimmomatic v. 0.36 (Bolger et al., 2014). Variant calling of each genotype was performed using freebayes v1.3.2 (Garrison and Marth, 2012). VCF output files of all samples were merged into a single VCF file using BCFtools and high quality SNPs with minimum allele frequency of 5% (QUAL > 30, AF > 0.05 and AF < 0.95, GQ > 20) were filtered using VCFtools 0.1.16 (Danecek et al., 2011). A maximum likelihood phylogeny was inferred based on the filtered SNPs using RAxML-NG v. 1.0.2-master (Kozlov et al., 2019) with 100 bootstrapping replications.

### FISH Experiments

Sixteen genotypes of *Ae. tauschii* were received from the Seeds and Plant Improvement Institute of Iran (SPII) or IPK gene bank of Germany (Supplementary Table 2) and analyzed by FISH. Oligo-(GAA)_10_ (Pedersen and Langridge, 1997), oligo-p*Ta*535-1 (Komuro et al., 2013; Tang et al., 2014), oligo-pSc119.2-1 and oligo-p*As*1-1 probes were used for karyotype analysis (Table 1). Oligo-(GAA)_10_ and oligo-pSc119.2-1 were directly labeled at the 5′ end with FAM (6-carboxyfluorescein) and oligo-p*Ta*535-1 and oligo-p*As*1-1 were 5′-end-labeled with TAMRA. Oligonucleotide probes were synthesized by Bioneer Co. Ltd. (Daejeon, South Korea). Synthesized probes were diluted using 1 × TE solution (pH 7.0). A partial sequence of the CRM repeat unit (3D:250158225-250159002 region) was amplified using forward: 5′AGGGCCTAGCTTTGAGAAGG, and reverse: 5′ATGGATATCGCTTTGGTGGA primers, labeled with a nick translation kit (Jena Bioscience, Jena, Germany), recovered by ethanol precipitation and used as a probe in FISH for localization of CRM elements. Chromosome preparation and FISH were performed according to Abdolmalaki et al. (2019), except that root tips pretreatment time with nitrous oxide (N_2_O) was reduced to 2 hours.

**TABLE 1:** Partial sequences of the identified *Ae. tauschii* satellite clusters homologous to the oligonucleotide probes used in the present study i.e., oligo-p*As*1-1, oligo-p*Ta*535-1, and oligo-p*Sc*119.2-1.

Cluster or probe	Sequence (5′ - > 3′)
CL1	**CCTTTCTGACTTCATTTGTTATTTTTCATGCATTTACTA** **ATTATTTTGAGCTATAAGAC**
**oligo-p*As*1-1**	**CCTTTCTGACTTCATTTGTTATTTTTCATGCATTTACTA** **ATTATTTTGAGCTATAAGAC**
CL57	**GAAACCCTGATACTCCGAAAGAGATTGTCCAGTTTGT** **ACACGAGGTGCGTCCAGTTTTC**
**oligo-p*Ta*535-1**	**GAAACCCTGATACTCCGAAAGAGTTTGTCCAATTTGT** **ACGTGACGTGCGTCAAGTTTTT**
CL211	**CCGTTTCGTGGACTATTACTCACTGTTTTGGGGTCCC** **GGAGTGAT**
**oligo-pSc119.2-1**	**CCGTTTTGTGGACTATTACTCACCGCTTTGGGGTCCC** **ATAGCTAT**

*Nucleotides different from the homologous sequence are highlighted in red. Oligo-pAs1-1, oligo-p*Ta*535-1, and oligo-p*Sc*119.2-1 sequences are originally from *Ae. tauschii*Rayburn and Gill, 1986, Triticum aestivum Komuro et al., 2013, and *Secale cereal*Bedbrook et al., 1980.*

## Results

### General and Intervarietal Repeatome Structure of *Ae. tauschii*

In the present study, publicly available raw Illumina 150 bp paired end reads from 29 different *Ae. tauschii* accessions belonging to *anathera*, *strangulata*, and *tauschii* subspecies (Supplementary Table 1) were analyzed using RepeatExplorer2 pipeline to elucidate the evolutionary patterns of highly repetitive sequences among subspecies. The GC content for *Ae. tauschii* genome showed a value of 47% and highly and moderately repetitive sequences constitute 77.35% of the nuclear genome (Supplementary Table 2). The majority (61.56%) of the repeats are composed of transposons with 58.42% of which being retrotransposons. On the other hand, class II transposons contributed to only 2.03% of the repeats. Long terminal repeat LTR retrotransposons are the most abundant mobile elements and composed 58.27% of the genome. LTRs divided into Ty3/gypsy and Ty1/copia super families with 38.65 and 19.35% of genome, respectively (Supplementary Table 3).

The proportion of the identified repeat clusters and the number of reads in each cluster (which is proportional to their genomic abundance) per accessions and other details including satellite probabilities and related indices, i.e., connected component index (C) and are pair completeness index (P) (Novák et al., 2017) are shown in Supplementary Table 4 where cluster numbers are in order of their amount in the genome. Comparative repeatome analysis revealed that the overall contents and proportions of the identified repetitive sequences are highly similar between the three subspecies of *Ae. tauschii* (Supplementary Figure 1 and Supplementary Table 4) except seven repeat clusters which showed significantly different abundances between the studied subspecies. The read counts of these differentially amplified repeats are presented in Supplementary Table 5, and results of their statistical comparisons between the subspecies are presented in Supplementary Table 6. Monomer, analysis of individual genomes using TAREAN (data not presented) showed that monomer sequences of tandem repeats are completely identical among all the *Ae. tauschii* genotypes.

**FIGURE 1:**
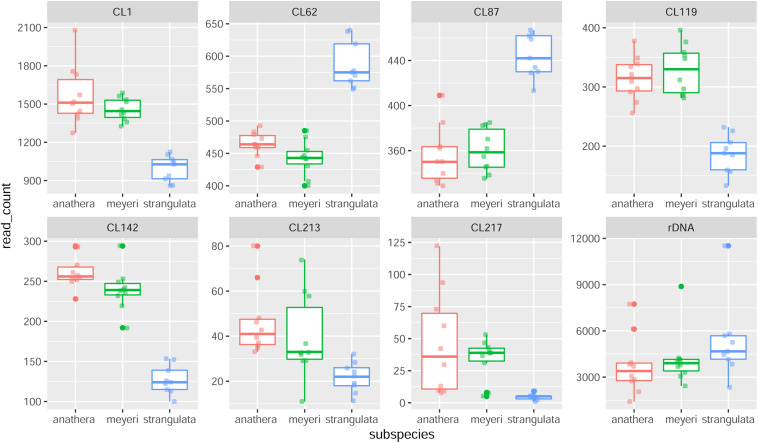
Box plot of repetitive DNA abundances (number of reads in each read sample) in 29 *Ae. tauschii* accessions belonging to subsp. *anathera* (red dots), subsp. *meyeri* (green dots) and subsp. *strangulata* (blue dots). CL119, CL1, CL142, CL87, and CL62 clusters showed significant differences between subspecies and CL213 and rDNA (45S) showed considerable variation between genotypes.

**FIGURE 2:**
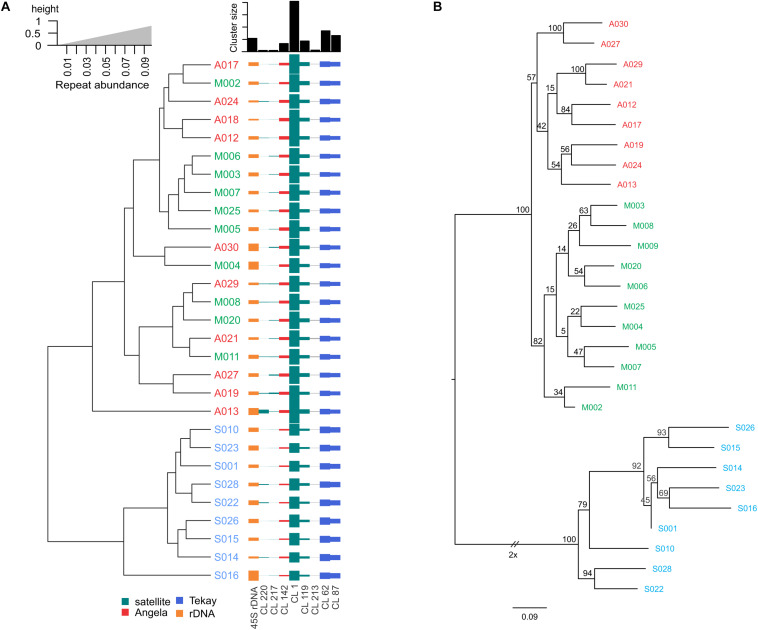
**(A)** Dendrogram of 29 *Ae. tauschii* accessions derived from the abundance of repetitive sequences differentially amplified between genotypes and subspecies as inferred from RepeatExplorer2. **(B)** Phylogenetic tree by maximum likelihood method based on SNPs discovered from low coverage Illumina reads. Numbers at the nodes are bootstrap values from 100 replications. Accession codes have been shown in red (for subsp. *anathera*), green (subsp. *meyeri*), and blue (subsp. *strangulata*).

**FIGURE 3:**
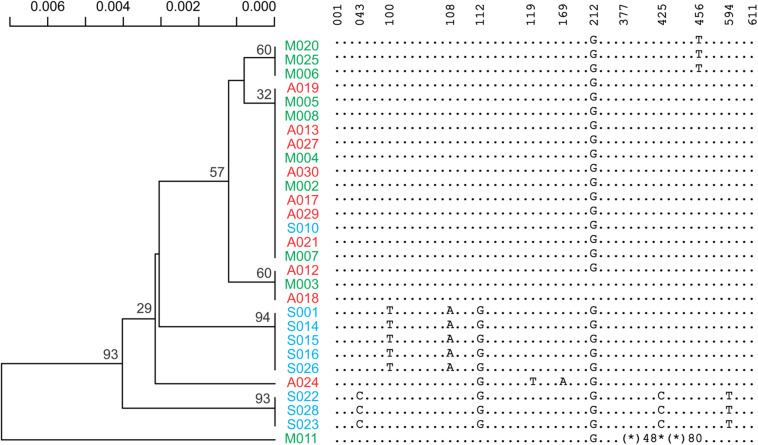
A maximum likelihood phylogenetic tree inferred from ITS1-5.8S-ITS2 part of rDNA sequences. Reduced form of the sequences covering all the variants and their locations has also been presented. An asterisk indicates deletion and an asterisk followed by a number indicates the number of deleted residues.

Sixteen different satellite repeats representing 3% of the genome of *Ae. tauschii* were identified with unit lengths ranging from 44 to 6371 nucleotides, although a majority (13) of them had unit length in range of 118 to 567 nucleotides. The proportion and other details of each of these tandem repeats including consensus length and satellite probability are shown in Supplementary Table 7. Seven clusters including CL1, CL62, CL87, CL213, CL217, CL119, and CL142 showed subspecies-specific amplification during the diversification of *Ae. tauschii* (Figure 1). CL62 and CL87 were Tekay retrotransposons belong to Ty3/gypsy super family and showed significantly higher abundances, while CL213, CL217, CL119, and CL1 tandem repeats and CL142 (Ty1/copia) retrotransposon showed significantly lower abundances in subsp. *strangulata* compared with subsp. *anathera* and subsp. *meyeri* (*p* value < 0.05; Supplementary Table 6). 45S rDNA abundances were highly variable between accessions but their abundances were not subspecies specific (Figure 1). Cluster CL220 was observed in only some of the studied accessions (Supplementary Table 5). Based on a dendrogram made from read counts of these clusters (Figure 2A), subsp. *strangulata* was clearly confined to a distinct clade. We extracted ITS1-5.8S-ITS2 sequences (Supplementary Table 8) from all the accessions and made a maximum likelihood tree (Figure 3) which could not resolve the subspecies, although most of the *strangulata* genotypes were grouped together. Compared with ITS sequences, it seems that repeat abundance is a more efficient tool for intraspecific classification in *Ae. tauschii*.

### Satellite Repeats Characterization

Homology search of the identified satellite repeats using BLASTn revealed that CL1 is homologous to p*Ta*-173 (GenBank: KC290893.1) (Komuro et al., 2013), p*As*1 (Rayburn and Gill, 1986) and Afa family (Nagaki et al., 1995). CL57 is homologous to p*Ta*-s53 (KC290895.1) and p*Ta*-535 (KC290894.1) that is also related to p*As*1. CL119 satellite is homologous to 4P6-2 (AY249987.1), a repeat that has been identified already by FISH using bacterial artificial chromosome (BAC) clones as probes (Zhang et al., 2004). CL23 has 78% identity with p*Ta*-451 (KC290912.1), CL173 is homologous to *Triticum aestivum* clone p*Ta*-465 sequence and CL211 is homologous to p*Ta*-835 (KC290898.1).

**FIGURE 4:**
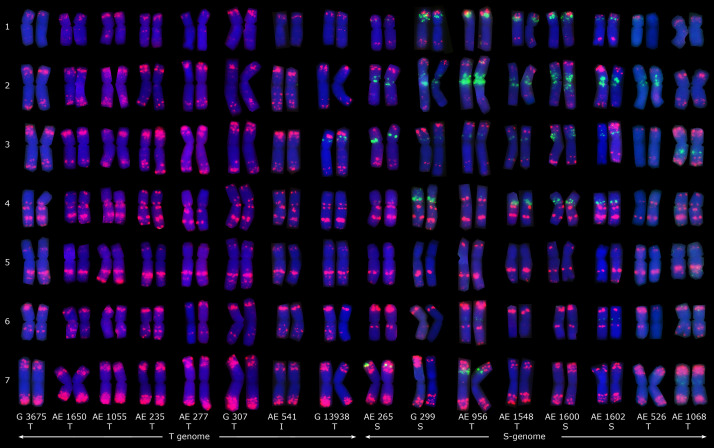
Distribution patterns of (GAA)_10_ (green) and pTa535-1 (red) probes on the mitotic metaphase chromosomes of sixteen *Ae. tauschii* accessions. Types of spike morphology (S, subsp. *strangulata*; T, subsp. *tauschii*; and I, intermediate) has also been indicated.

**FIGURE 5:**
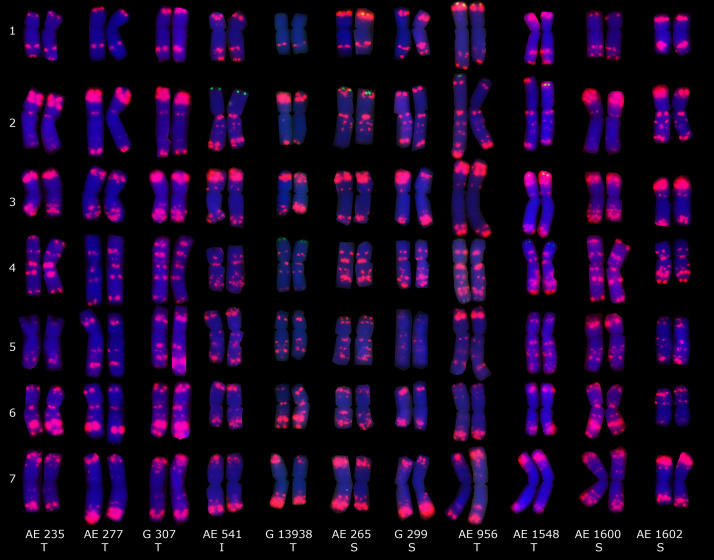
Distribution patterns of pAs1-1 (red) and oligo-pSc119.2-1 probes (green) on the mitotic metaphase chromosomes of eleven *Ae. tauschii* accessions. Spike morphologies of S, T, and I refers to as subsp. *strangulata*, subsp. *tauschii*, and intermediate form, respectively.

Fluorescence *in situ* hybridization using oligo-p*As*1-1 (homologous to the most abundant tandem repeat CL1), oligo-p*Ta*535-1 (homologous to CL57 tandem repeat), oligo-pSc119.2-1 (homologous to CL211 tandem repeat) and (GAA)_10_ was applied on sixteen *Ae. tauschii* genotypes (Figures 4, 5). Oligo-p*As*1-1 and oligo-p*Ta*535-1 probes generally produced comparable patterns, and have been widely used for the identification of D genome chromosomes (Tang et al., 2014). Oligo-p*Sc*119.2-1 probe generated weak signals in subtelomeric regions of chromosome arms 1DS, 2DS, 3DS, and 4DS (Figure 5) in some accessions. Although none of the probes discriminated subspecies (GAA)_10_, signal patterns generated two karyotype groups, that poorly agreed with botanical classification (Figure 4).

### Distribution Patterns of Major Retrotransposons on Chromosomes

**FIGURE 6:**
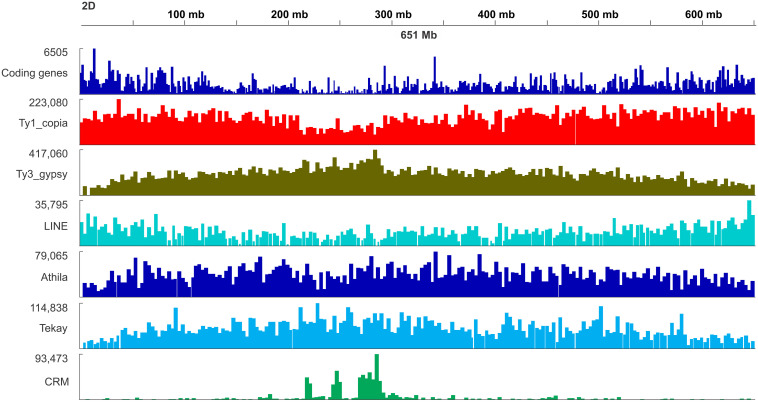
Distribution map of genes and selected retrotransposon lineages including Ty1/copia, Ty3/gypsy, LINE, Athila, Tekay, and CRM retrotransposons along the chromosome 2D of *Ae. tauschii*.

**FIGURE 7:**
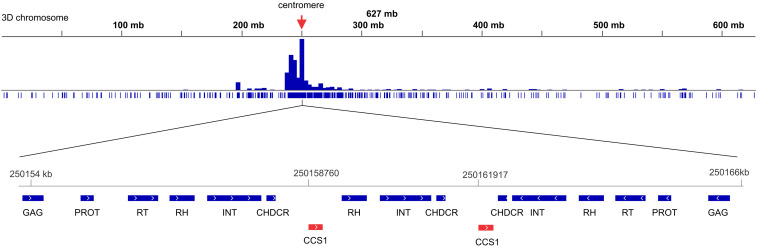
Density of CRM lineage of Ty3/gypsy retrotransposon along the chromosome 3D of *Ae. tauschii*. A small part from the centromeric region which is highly enriched in CRM elements has been show in detail. BLASTn mapped the Triticeae specific CCS1 centromeric repeat (red elements) between the CRM elements. CHDCR, chromodomain of centromeric retrotransposons; INT, integrase; PROT, protease; RH, ribonuclease H; RT, reverse transcriptase.

**FIGURE 8:**
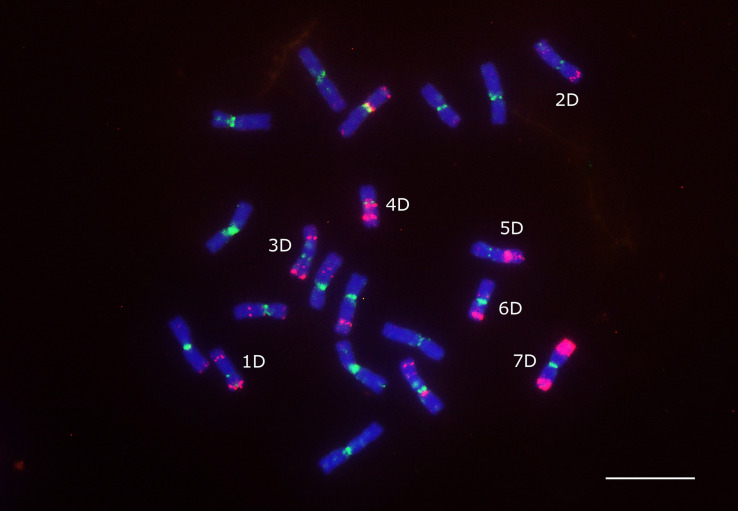
Localization of CRM element (green signals) on the mitotic chromosomes of an F_1_ hybrid from a cross between emmer wheat (*T. dicoccum* “TazeabadAliabad”) and *Ae. tauschii* “G 299.” Oligo-pTa535-1 probe (red signals) was also applied to identify the D genome chromosomes. Scale bar = 10 μm.

The density of the Ty1/Copia and LINE (short interspersed nuclear element) superfamilies accompanied codding gene density and increased from the centromere toward the telomere whereas the density of the Ty3/Gypsy superfamily and two of its most abundant lineages, i.e., Athila and Tekay increased in the opposite direction (Figure 6). The centromere-specific retrotransposon CRM (homologous to *cereba*) that is a lineage of Ty3/gypsy chromoviruses has been preferentially accumulated in centromeres (Nagaki et al., 2003). BLASTn mapped the previously identified Triticeae specific CCS1 centromeric repeat (Aragon-Alcaide et al., 1996) between the CRM elements indicating that these elements are enriched for the centromere core sequences (Figure 7). The CRM peaks showed uneven distribution within the 7 *Ae. tauschii* chromosomes (Figure 7). We used root tips of an F_1_ hybrid generated from a cross between emmer wheat and *Ae. tauschii* ‘G 299’ for the localization of CRM elements: Chromosomes 6D and 7D showed stronger signals relative to the other D genome chromosomes (Figure 8).

### SNP Calling and Genetic Diversity Analysis

The same Illumina reads that used for the repeat identification, were also used for variant calling and SNP identification among the *Ae. tauschii* accessions. A total of 466,498 SNPs identified from all the genotypes after filtering. Distributions of each type of SNP were as follows: A/G, 147010 (31.1%), C/T, 149165 (32%), A/T, 35523 (7.6%), A/C, 44284 (9.4%), C/G, 46079 (9.8%), and G/T, 44437 (9.5%). Of the 466498 identified SNPs, 296175 (63.4%) were classified as transitions (A/G or C/T), and 170323 (36.5%) were classified as transversions (A/T, A/C, C/G, and G/T) (Supplementary Table 9). A phylogenetic tree based on the identified SNPs and Nei’s genetic distances was constructed, based on which, almost all *anathera* and *strangulata* genotypes were grouped according to their subspecies (Figure 2B). Genotypes of var. *meyeri* showed a substantial genetic diversity based on SNP analysis. The SNP-derived phylogenetic tree rather supported the tree generated from the abundances of subspecies and genotype specific clusters (Figure 2B). This agreement was specifically obvious for *strangulata* subspecies whose genotypes were clustered together in both trees, although the tree of repeat abundances was unable to unify genotypes belonging to *anathera* subspecies.

## Discussion

Genomic repetitive sequences commonly show species-specific sequence type, abundance, and distribution patterns, however, their intraspecific variations have been poorly described. To see how the repeatome differentiate in *Ae. tauschii*, we used publicly available genomic Illumina read data and compared the repetitive sequences between 29 *Ae. tauschii* genotypes from different subspecies, i.e., *anathera*, *meyeri*, and *strangulata* using RepeatExplorer (Novák et al., 2020).

Repetitive DNA constitutes about 80% of angiosperm genomes with 1C DNA value greater than 5.0 pg (Flavell et al., 1974). Nearly, 85% of the maize (Schnable et al., 2009), wheat (Appels et al., 2018), and barley (Mayer et al., 2012) genomes are composed of transposable elements, the majority of which are LTR retrotransposons. Our analysis using RepeatExplorer2 showed that the repetitive sequences constitute 77.35% of the nuclear genome of *Ae. tauschii*, the majority of which (61.56%) are composed of transposons with 58.42% of which being retrotransposons (Supplementary Table 3). LTR retrotransposons were found to be the most abundant mobile elements and composed 58.27% of *Ae. tauschii* genome. The LTR retrotransposons divided to Ty3/gypsy and Ty1/copia super families with 38.65 and 19.35% of genome, respectively. Estimation of the amount of transposable element by RepeatExplorer is in agreement with the amount of transposable element (55.12%) estimated via *Ae. tauschii* genome sequencing (Jia et al., 2013). On the contrary, class II transposons contributed to only 2% of the repeats. This estimate was not in agreement with 11% (Jia et al., 2013) and 16% (Luo et al., 2017) ratios estimated by genome sequencing projects of *Ae. tauschii*. A similar proportion of 2–3% class II transposons is found in each of maize (Meyers et al., 2001) and *Arabidopsis* (The_Arabidopsis_Genome_Initiative, 2000) whose genomes are 1.9 and 16.8 times smaller than that of *Ae. tauschii* (4,968 Mbp), respectively. However, this estimate is different from that of *Brassica* and rice, whose genomes contain ∼6 and 12% class II DNA transposons, respectively (Jiang and Wessler, 2001; Jiang et al., 2004).

Sixteen different satellite repeats representing 3% of the *Ae. tauschii* genome were identified. The unit length of the identified satellites ranged from 44 to 6371 nucleotides, although a majority (thirteen) of these had a unit length in the range of 118 to 567 nucleotides. The unit length of most tandem repeat families in plants varies from 150 to 180 bp, but can reach up to 1000 bp or more (Melters et al., 2013). For example, centromeric tandem repeats lengths are 178 bp in Arabidopsis (Copenhaver et al., 1999), 155 bp in rice, and 156 bp in maize (Ananiev et al., 1998; Melters et al., 2013), a length sufficient for wrapping around a single nucleosome (Henikoff et al., 2001).

Unequal crossing-over and strand slippage are the mechanisms which can easily explain the duplication of tandem repeats in the genome (Garrido-Ramos, 2015). Tandem repeats are highly prevalent at centromeres of both the animal and plant genomes (Melters et al., 2013); however, here the most frequent satellites such as CL1 and CL57 are distributed outside the centromeres and toward the distal end of chromosome arms. CL1, CL34, and CL57 are homologous to the already identified repeats p*As*1 or Afa family (Nagaki et al., 1995; Komuro et al., 2013), p*Ta*-451 (Komuro et al., 2013), and p*Ta*535 (Komuro et al., 2013), respectively.

A key result of this study is that a few repetitive sequence clusters were revealed to be differentially proliferated between *Ae. tauschii* subspecies. Although the type and amount of the identified repeats were generally the same between accessions, however, we found seven repeats with differential amplification between subspecies (Figure 1 and Supplementary Table 7). In a dendrogram generated based on the abundances of these repeats, subsp. *strangulata* differed from subsp. *meyeri* and subsp. *anathera* while the latter two were grouped together. Taken together, these results are in agreement with the monotypic nature, distinct spike morphology, and the lower geographic dispersal and genetic diversity of subsp. *strangulata* compared with the other subspecies, e.g., subsp. *tauschii* and intermediate forms (Kihara and Tanaka, 1958; Dvorak et al., 1998).

Variation in repeat abundance is common during the speciation and might change DNA C value. For example, differential lineage-specific amplification of transposable elements has been observed in *Gossypium* (Hawkins et al., 2006). Subspecific repeats amplification has also been reported in other plants. In *Beta nana* copy number of a specific satellite, was more than tenfold higher than in *B. lomatogona* and up to 200 times higher than in *B. vulgaris*, indicating the different levels of sequence amplification during evolution in the genus *Beta* (Kubis et al., 1997). In rice, the different repetitive sequence families have been differentially amplified between *indica* and *japonica* rice (Ohmido et al., 2000).

Our study suggests that repetitive sequence abundances could provide additional helpful data for phylogenetic and genome evolution studies. Comparative graph-based clustering of next-generation sequence reads has been utilized for the phylogenetic analysis. It has been shown that the abundance of repetitive elements has a phylogenetic signal and can be used as a continuous character to infer phylogenetic trees (Dodsworth et al., 2015a,b). CL220 tandem repeat and 45S rDNA abundances were highly variable between genotypes but their abundances were not subspecies specific. Variation in rDNA copy number between individuals within a species is well documented (Rogers and Bendich, 1987). Variation in rDNA copy number is thought to be tolerated because of redundancy, and the observation that only a subset of the repeats is transcribed at any one time (McStay and Grummt, 2008; Lopez et al., 2021).

Besides the repeat abundances, we further used a reference-based SNP calling and ITS1-5.8S-ITS2 sequences (Supplementary Table 8) for phylogeny of *Ae. tauschii* accession. The ITS tree did not group subspecies even the *strangulata* accessions together (Figure 3), implying lack of ITS sequence efficacy for intervarietal classification. A near complete differentiation of *anathera* and *strangulata* subspecies was observed using SNP analysis although var. *meyeri* showed a higher genetic diversity (Figure 2B). There are reports that some *meyeri* accessions, specifically those from the west coast of the Caspian Sea, are genetically closer to *strangulata* (Lubbers et al., 1991; Dvorak et al., 1998; Wang et al., 2013). Although phylogenetic analysis using the repeat abundances placed all the *strangulata* accessions in a distinct clade but could not discriminate between *anathera* and *meyeri* (Figure 2).

Providing that *anathera* and *meyeri* varieties be considered as a single *tauschii* subspecies as suggested basically by the botanical classifications, we can conclude that subspecific differential amplification of CL62 and CL87 and CL217 (all belonging to Ty3/gypsy super family) and CL142 (belonging to Ty1/copia super family) retrotransposons have been resulted from change in their activity after *strangulata* subsp. divergence. In fact, LTR retrotransposons are the most dynamic part of the genome, and an important source of within species differences in repeat abundances (Vitte and Panaud, 2005).

Our results suggest the involvement of repeat amplification rates in botanical differences such as spike morphology between *Ae. tauschii* genotypes. Types and abundances of repetitive DNA might have an impact on the expression of the adjacent genes (Ramírez-González et al., 2018; Bariah et al., 2020). TEs also have an impact on DNA methylation and expression of nearby genes in the different plant species (Makarevitch et al., 2015; Wang et al., 2018; Stritt et al., 2020). TEs in gene promoter might affect gene expression in a tissue-specific manner as cis-regulatory elements or through other epigenetic mechanisms (Ramírez-González et al., 2018). For example, MITE domestication into miRNA precursors might have an important role in gene expression in wheat (Poretti et al., 2020). Association between a specific TE insertion into a gene and the levels of gene expression in wheat has also been reported (Domb et al., 2019). TE insertions can also have a direct effect on phenotypes such as brittle rachis and heading date in wheat (Jiang et al., 2019; Shi et al., 2019). Various functions ranging from chromosome organization and pairing to the modulation of gene functions are also proposed for tandem repeats (Martienssen, 2003; Kloc and Martienssen, 2008; Garrido-Ramos, 2015).

Fluorescence *in situ* hybridization using p*As*1-1 (homologous to CL1), p*Ta*535-1 (homologous to CL57), p*Sc*119.2-1 (homologous to CL211), and (GAA)_10_ oligo-nucleotide probes could not discriminate *Ae. tauschii* subspecies but (GAA)_10_ signal patterns generated two distinct karyotype groups. The two karyotype groups were poorly agreed with botanical classification of subspecies, but were concurrent with the molecular marker-based phylogeny that proposed the presence of two distinct lineages of *Ae. tauschii* (Dvorak et al., 1998; Mizuno et al., 2010; Wang et al., 2013). This is not the first report on *Ae. tauschii* that links karyotype to genetic structure. The presence of two distinct genomes in *Ae. tauschii* has already been demonstrated as well based on the GAA distribution patterns (Zhao et al., 2018). Based on the spike morphology and karyotypic analysis, Zhao et al. (2018) concluded that subsp. *tauschii* var. *meyeri*, as an intermediate form, should be redesignated subsp. *strangulata* var. *meyeri*. The FISH pericentromeric signal resulting from (GAA)_10_ probes on chromosome 4 seems to be specific to *strangulata* subspecies and is not available on the other two types (Zhao et al., 2018), but chromosome 4 of “AE 956” and “AE 1548”– which belong to *tauschii* subspecies, exceptionally showed a very weak (GAA)_10_ signal in this study (Figure 4).

## Conclusion

Although the SNP-based analysis proved to be the gold standard for the intraspecific classification, the present study demonstrated that different classes of repetitive DNA sequences have differentially accumulated between *tauschii* and *strangulata* subspecies of *Ae. tauschii*. The differential repeat amplifications generally agreed with morphological differences. Taken together, the results imply that repeatome is differentially evolved even among highly closely related lineages.

## Data Availability Statement

The original contributions presented in the study are included in the article/Supplementary Material; further inquiries can be directed to the last corresponding author.

## Author Contributions

RE and FO assisted in FISH experiments. GM and PM conceived and designed the research. GM conducted bioinformatics analysis, did FISH experiments, and wrote the manuscript. All authors contributed to the article and approved the submitted version.

## Conflict of Interest

The authors declare that the research was conducted in the absence of any commercial or financial relationships that could be construed as a potential conflict of interest.

## Publisher’s Note

All claims expressed in this article are solely those of the authors and do not necessarily represent those of their affiliated organizations, or those of the publisher, the editors and the reviewers. Any product that may be evaluated in this article, or claim that may be made by its manufacturer, is not guaranteed or endorsed by the publisher.
