# Supplementary material for: Differentially Amplified Repetitive Sequences Among Aegilops tauschii Subspecies and Genotypes
Source: Front Plant Sci. 2021 Aug 19;12:716750. doi: 10.3389/fpls.2021.716750 (PMC8417419; doi:10.3389/fpls.2021.716750)
Supplement: Supplementary file 1 [file Data_Sheet_1.zip › Supplementary Figure 1.docx]

Differentially amplified repetitive sequences among *Aegilops tauschii* subspecies and genotypes

**Figure S1.** Composition of abundance of repetitive sequences in 29 *Ae. tauschii* genotypes belonging to *anathera*, *strangulata* and *tauschii* subspecies as revealed using RepeatExplorer2. The size of the rectangles is proportional to the number of reads in a cluster for each genotype. For a number of the clusters, the number of reads differs considerably among different subspecies and genotypes. Accession codes has been shown in red (for ssp. *anathera*), green (ssp. *meyeri*) and blue (ssp. *strangulata*).
